# The impact of anxiety on gait impairments in Parkinson’s disease: insights from sensor-based gait analysis

**DOI:** 10.1186/s12984-024-01364-3

**Published:** 2024-04-30

**Authors:** Xiaodan Zhang, Yulan Jin, Mateng Wang, Chengcheng Ji, Zhaoying Chen, Weinv Fan, Timothy Hudson Rainer, Qiongfeng Guan, Qianyun Li

**Affiliations:** 1 Department of Neurology, Ningbo NO.2 Hospital, Ningbo, Zhejiang Province China; 2 Department of Emergency Medicine, University of Hong Kong, Hong Kong SAR, China; 3 Department of Clinical Laboratory, Ningbo NO.2 Hospital, Ningbo, Zhejiang Province China; 4 Department of General Surgery, Yinzhou NO.2 Hospital, Ningbo, Zhejiang Province China; 5 School of Medicine, Shaoxing University, Shaoxing, Zhejiang Province China

**Keywords:** Gait analysis, Parkinson’s disease, Anxiety, Wearable sensors, Treatment

## Abstract

**Background:**

Sensor-based gait analysis provides a robust quantitative tool for assessing gait impairments and their associated factors in Parkinson’s disease (PD). Anxiety is observed to interfere with gait clinically, but this has been poorly investigated. Our purpose is to utilize gait analysis to uncover the effect of anxiety on gait in patients with PD.

**Methods:**

We enrolled 38 and 106 PD patients with and without anxiety, respectively. Gait parameters were quantitively examined and compared between two groups both in single-task (ST) and dual-task (DT) walking tests. Multiple linear regression was applied to evaluate whether anxiety independently contributed to gait impairments.

**Results:**

During ST, PD patients with anxiety presented significantly shorter stride length, lower gait velocity, longer stride time and stance time, longer stance phase, smaller toe-off (TO) and heel-strike (HS) angles than those without anxiety. While under DT status, the differences were diminished. Multiple linear regression analysis demonstrated that anxiety was an independent factor to a serials of gait parameters, particularly ST-TO (B = -2.599, (-4.82, -0.38)), ST-HS (B = -2.532, (-4.71, -0.35)), ST-TO-CV (B = 4.627, (1.71, 7.64)), ST-HS-CV(B = 4.597, (1.66, 7.53)), ST stance phase (B = 1.4, (0.22, 2.58)), and DT stance phase (B = 1.749, (0.56, 2.94)).

**Conclusion:**

Our study discovered that anxiety has a significant impact on gait impairments in PD patients, especially exacerbating shuffling steps and prolonging stance phase. These findings highlight the importance of addressing anxiety in PD precision therapy to achieve better treatment outcomes.

**Supplementary Information:**

The online version contains supplementary material available at 10.1186/s12984-024-01364-3.

## Introduction

As a prevalent neurodegenerative disorder, Parkinson’s disease (PD) affects millions of individuals globally each year [1]. Its incidence and prevalence increased at a remarkable rate, making it the fastest growing neurological disorder worldwide [2]. PD significantly affects patients’ daily lives and places a heavy burden on families, caregivers, and society [2]. Gait impairment is a particularly prominent motor symptoms in all PD stages. Specifically, during the prodromal phase of PD, patients typically experience an asymmetrical interlimb movement and slightly reduced gait velocity and stride length [3]. As the disease progresses, gait changes become more pronounced, emerging bradykinesia, shuffling steps, fragmented turns, and gait initiation problems [3]. During advanced stage, freezing of gait and festination become more frequent, with increased risk of fall and necessities for assistance devices (e.g., wheelchairs) [3]. A systematic review of 66 studies found that the weighted prevalence of freezing of gait was 37.9% in early-stage PD and 64.6% in advanced PD [4]. Another study showed that nearly two-thirds of patients experienced falls within 7 years of follow-up, leading to an increased risk of fractures and a sedentary lifestyle [5]. A summary of 29 studies concluded that gait impairments affected mostly on quality of life among all motor symptoms [6]. Therefore, it is crucial to develop productive strategies to manage gait disturbances to improve quality of life in PD patients.

PD patients additionally suffered with a range of neuropsychological problems. Anxiety is a common emotional problem that affects up to a quarter of PD patients [7]. During clinical practice, we observed that the gait disturbance in PD patients with anxiety tend to be more marked. Studies have demonstrated that anxiety exacerbated freezing of gait [8]. However, the impact of anxiety on most other gait impairments in PD patients is poorly investigated.

Sensor-based gait analysis brings an objective and quantitative assessment of gait disturbances, allowing for a deeper insight into identification, progression, and therapy management of PD. For instance, a longitudinal prospective study on 696 healthy patients with PD risk factors found that quantitative gait parameters could identify early PD and its progression within the prodromal phase [9]. Another study demonstrated that these parameters, such as gait velocity, stride length, toe-off angle (TO), and their variabilities, provide an objected value for differentiating atypical Parkinsonian disorders from idiopathic PD [10]. Sensor-based gait parameters could be utilized as markers of PD progression as well [11]. On therapy management, Curtze used inertial sensors to monitor PD patients’ responsiveness to L-dopa [12]. Sensor-based technology were also applied in the training of patients with gait and balance disorders [13]. Compared with traditional visual observation and subjective evaluation, this technology reflects subtle changes that may not be noticeable to observers, collects data simultaneously on multiple parameters, and produces a general overview of gait performances [3]. Consequently, it is a valuable tool to improve the assessment and management of PD, supplying objective and accurate data that can assist clinical decision-making.

The objective of this study was to evaluate the potential impact of anxiety on gait through quantitatively analyzing gait parameters in PD patients with and without anxiety. Additionally, our research intends to provide a theoretical basis for future randomized controlled trials (RCTs) that aim to determine whether intervention of anxiety symptoms could result in an improvement of gait performance among PD patients.

## Methods

### Participants

Consecutive PD patients were enrolled in a prospective cross-sectional study at Ningbo NO.2 Hospital during September 2019 to December 2021. The inclusion criteria were: (1) patients met MDS clinical diagnostic criteria for PD [14]; (2) patients could walk independently; (3) recent symptoms and medication of patients were stable. The exclusion criteria were: 1) patients with other diseases that might impact gait and balance; (2) patients unable to accomplish the doctor’s orders. The study was conducted in compliance with the Helsinki Declaration. All participants have signed a consent form. Ethical approval was granted by Ningbo NO.2 Hospital Institutional Review Board (Approval number: PJ-NBEY-KY-2020-023-01).

### Clinical data collection

The demographic and medical data of all participants were collected. A neurologist performed physical examinations and assessed the severity of PD according to the motor section of the Unified Parkinson’s Disease Rating Scale (UPDRS-III). The Hamilton Anxiety Scale (HAMA) was used for assessment of anxiety and Hamilton Depression Rating Scale-24 (HAMD) was used for evaluation of depression. For HAMA, scores of 0–7 are considered normal, while scores of 8–13 indicate possible anxiety, 14–20 anxiety, 21–28 obvious anxiety, and 29 and above severe anxiety. According to these criteria, patients with scores 14 and above were considered to have anxiety. The Mini-Mental State Examination (MMSE) was applied to evaluate cognitive function. All PD patients were evaluated in the OFF state, which was after the antiparkinsonian medication was stopped for 18 h.

### Gait evaluation

Gait data was collected through the JiBuEn gait analysis system [15]. This system consisted of shoes and modules of Micro-Electro-Mechanical System sensors that were installed in the heel bottoms of the shoes, lower leg, thigh, and waist. The motion data was collected by these sensors and finally transmitted to a computer.

Two walking tests were required for all participants, which was described in our previous study [15]: (1) single-task (ST) walking test: All participants walked on a straight line in a 10 m footpath at their preferred “natural” gait velocity, and gait parameters were collected during natural walking; (2) dual-task (DT) walking test: All participants walked according to the same straight line and simultaneously counted 100 backwards with their attention focusing on both tasks. A practice was given to each participant before the actual test.

**Fig. 1:**
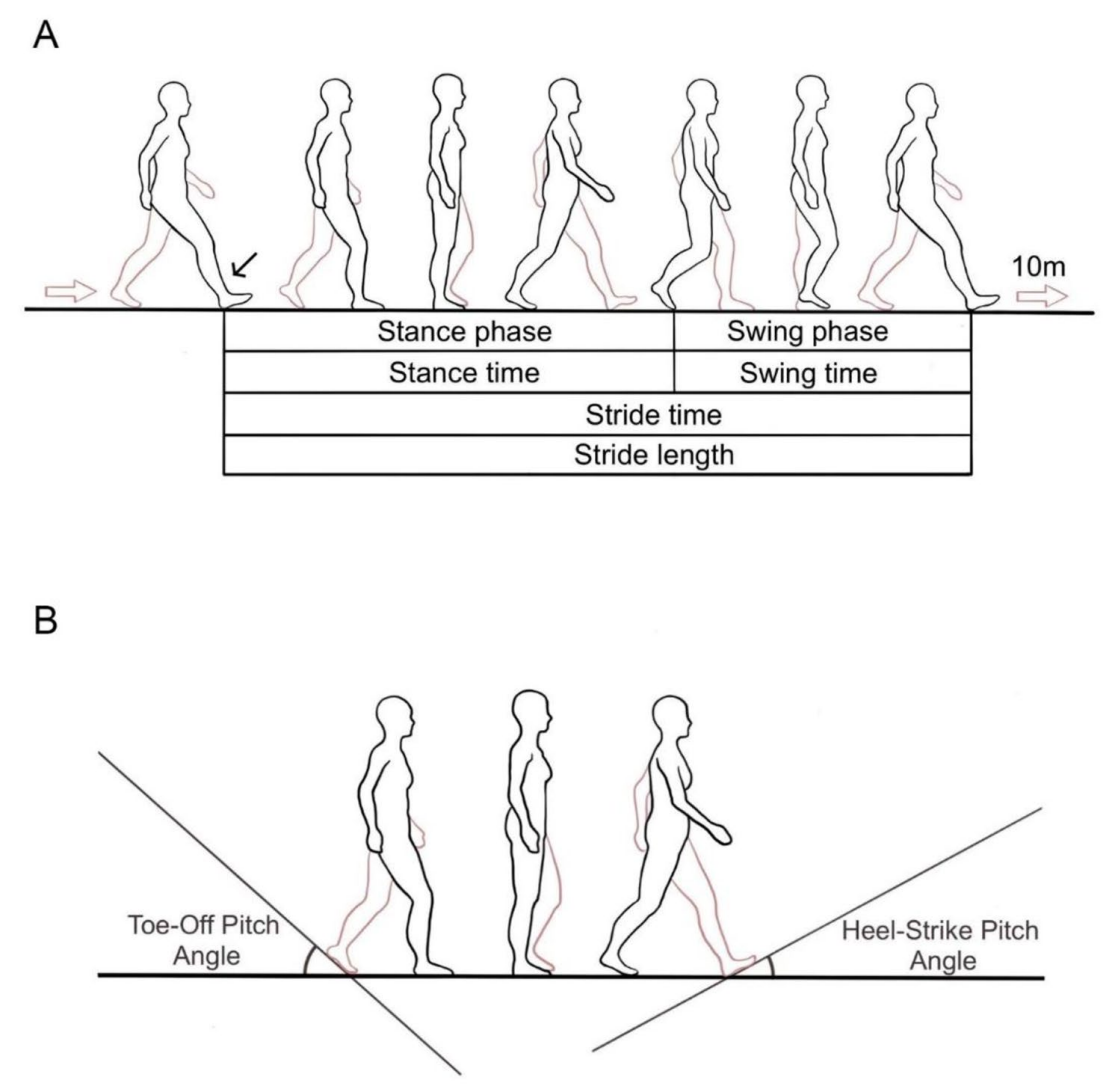
Illustration of gait parameters

At the same time, various gait parameters were obtained based on at least 40 steps, including stride length, gait velocity, stride time, stance time, and swing time (Fig. 1A) [15]. The stance phase (%) and swing phase (%) were calculated as the percentage of the stance and swing time in a step, respectively (Fig. 1A). Cadence (steps/min) was calculated as how many steps participants walked in a minute. Regarding to kinematic parameters, TO and heel-strike angle (HS) were detected (Fig. 1B). The coefficient of variation (CV) was calculated from the variability of all parameters, consisting of variability of stride length (stride-length-CV), gait velocity (gait-velocity-CV), stride time (stride-time-CV), swing time (swing-time-CV), stance time (stance-time-CV), TO (TO-CV), and HS (HS-CV) [16]. The asymmetry index (AI) was calculated as an indicator of the asymmetry of left and right side, including the asymmetry of stride length (stride-length-AI), gait velocity (gait-velocity-AI), stride time (stride time-AI), stance time (stance-time-AI), and swing time (swing-time-AI) [17]. The data was processed in a de-identified or pseudonymized state, and its processing was carried out with suitable safeguards in place.

### Statistical analysis

In this study, R 4.0.3 software was used for statistical analysis. For continuous (quantitative) data, the Shapiro normality test was used to determine the normality of data. Normally distributed continuous data was presented as mean ± standard deviation (SD) and comparison between two groups was conducted by independent t-test. Non-normally distributed continuous data was displayed as medians (interquartile ranges, IQRs) and the comparison between two groups was performed by the Wilcox test. Categorical data were statistically described by frequency (percentage), and comparison between groups was performed by chi-square test or Fisher’s exact test. When the bilateral p value was less than 0.05, the difference was considered statistically significant. Benjamini-Hochberg corrections were performed to adjust for multiple testing with a level of false discovery rate at 0.05.

We performed multiple linear regression analysis using *Lm()* function. Various gait parameters were dependent variables. Anxiety, age, gender, height, weight, disease duration, UPDRS-III score, MMSE score, and HAMD score were independent variables. *stepAIC()* function (R software, MASS package) was used to screen independent variables. When the independent variable was category variable, the minimum value group was used as the reference group. When the independent variable was a continuous variable, the continuous variable would be directly put into the linear regression model.

## Results

### Demographic characteristics

A total of 144 PD patients were included, consisting of 38 patients with anxiety and 106 patients without anxiety. There were more female patients in the anxiety group than in the non-anxiety group (*p* = 0.002). Compared with the non-anxiety group, the anxiety group displayed significantly longer disease duration (*p* = 0.001), higher HAMA and HAMD scores (*p* < 0.001), and slightly lower MMSE scores (*p* = 0.009). Table 1 shows the clinical characteristics of all patients.

**Table 1:** Clinical Characteristics of participants

Variable	Non-anxiety (*n* = 106)	Anxiety (*n* = 38)	*P*
Age (IQR)	67.5 (11.75)	64 (11.25)	0.255
Gender			**0.002**
Female (%)	39 (36.79%)	25 (65.79%)	
Male (%)	67 (63.21%)	13 (34.21%)	
Height, m (SD)	1.64 ± 0.08	1.63 ± 0.08	0.68
Weight, kg (IQR)	60 (14)	59 (10.62)	0.304
Disease duration, month (IQR)	36 (49.25)	72 (84)	**0.001**
UPDRS-III (SD)	34.14 ± 16.44	38.68 ± 14.04	0.132
HAMA (IQR)	5 (6)	17 (8)	**< 0.001**
HAMD (IQR)	6 (6.75)	18 (12.5)	**< 0.001**
MMSE (IQR)	27 (6)	25 (5)	**0.009**

Bold values highlight the significant difference; UPDRS-III, the Unified Parkinson’s Disease Rating Scale part III; HAMA, Hamilton Anxiety Scale; HAMD, Hamilton Depression Rating Scale-24; MMSE, Mini-Mental State Examination

### Gait performance in PD patients with and without anxiety

During ST, PD patients with anxiety presented significantly shorter stride length (*P* < 0.05), lower gait velocity (*P* < 0.001), longer stride time (*P* < 0.05) and stance time (*P* < 0.05), longer stance phase (*P* < 0.001), smaller TO (*P* < 0.05) and HS (*P* < 0.05) than those without anxiety, as shown in Table 2. There was no significant difference on swing time between the two groups during ST. Regarding gait variability and asymmetry, most parameters displayed no statistical significance between the two groups except for variability of TO (*P* < 0.05) and HS (*P* < 0.05).

**Table 2:** Comparison of gait parameters in patients with and without anxiety during single-task walking test

Variable	Non-anxiety (*n* = 106)	Anxiety (*n* = 38)	*P*	Adj.*P*
ST-stride-length (m)	0.99 ± 0.18	0.88 ± 0.18	**0.003**	**0.011**
ST-gait-velocity (m/s)	0.89 ± 0.19	0.75 ± 0.19	**< 0.001**	**< 0.001**
ST-stride-time (s)	1.12 (0.11)	1.18 (0.22)	**0.019**	**0.038**
ST-stance-time (s)	0.72 (0.1)	0.77 (0.17)	**0.007**	**0.018**
ST-swing-time (s)	0.4 ± 0.03	0.4 ± 0.04	0.551	0.827
ST-stance-phase (%)	64.43 (3.37)	65.7 (4.09)	**< 0.001**	**< 0.001**
ST-TO (°)	40.72 ± 6.87	37.55 ± 6.91	**0.016**	**0.036**
ST-HS (°)	26.6 ± 6.77	22.69 ± 6	**0.002**	**0.009**
**Variability and Asymmetry**				
ST-stride-length-CV (%)	22.22 (6.26)	23.3 (5.17)	0.223	0.401
ST-stride-length-AI (%)	22.78 (10.84)	21.98 (12.86)	0.734	0.881
ST-gait-velocity-CV (%)	27.3 (8.17)	28.33 (9.93)	0.66	0.878
ST-gait-velocity-AI (%)	31.91 (17.86)	32.17 (16.17)	0.971	0.971
ST-stride-time-CV (%)	21.05 (7.53)	20.95 (6.85)	0.888	0.94
ST-stride-time-AI (%)	18.46 (12.21)	18.47 (12.76)	0.683	0.878
ST-stance-phase-CV (%)	15.1 (3.47)	14.87 (3.38)	0.803	0.903
ST-stance-phase-AI (%)	10.29 (4.38)	10.27 (5.01)	0.303	0.496
ST-TO-CV (%)	18.21 (6.71)	22.18 (10.92)	**0.002**	**0.009**
ST-HS-CV (%)	25 (7.55)	28.36 (7.66)	**0.004**	**0.012**

Bold values highlight the significant differences; Adj.P: Benjamini-Hochberg corrected *p* value; ST: single-task walking test; CV: coefficient of variation; AI: asymmetry index; TO: toe-off angle; HS: heel-strike angle

When PD patients with anxiety were under DT walking, compared with patients without anxiety, the stride length was also significantly shorter (*P* < 0.05), the gait velocity was lower (*P* < 0.05), and the stance phase was higher (*P* < 0.05) (Table 3). Some of the significant differences existing in ST status (i.e. stride time, stance time, TO, HS, TO-CV and HS-CV) were not present during DT status.

**Table 3:** Comparison of gait parameters in patients with and without anxiety during dual-task walking test

Variable	Non-anxiety (*n* = 106)	Anxiety (*n* = 38)	*P*	Adj.*P*
DT-stride-length (m)	0.96 ± 0.21	0.84 ± 0.2	**0.002**	**0.018**
DT-gait-velocity (m/s)	0.95 ± 0.21	0.83 ± 0.2	**0.002**	**0.018**
DT-stride-time (s)	1.19 (0.22)	1.27 (0.34)	0.157	0.353
DT-stance-time (s)	0.78 (0.17)	0.87 (0.27)	0.066	0.201
DT-swing-time (s)	0.42 (0.05)	0.4 (0.05)	0.655	0.78
DT-stance-phase (%)	65.97 (4)	67.1 (4.94)	**0.008**	**0.048**
DT-TO (°)	39.52 (11.79)	37.33 (10.89)	0.067	0.201
DT-HS (°)	24.83 ± 7.42	22.01 ± 6.29	**0.039**	0.176
**Variability and Asymmetry**				
DT-stride-length-CV (%)	23.38 (9.66)	24.58 (6.32)	0.399	0.618
DT-stride-length-AI (%)	25 (17.02)	28.06 (11.7)	0.565	0.726
DT-gait-velocity-CV (%)	31.58 (12.26)	30.65 (7.48)	0.724	0.78
DT-gait-velocity-AI (%)	38.03 (25.52)	36.3 (17.34)	0.737	0.78
DT-stride-time-CV (%)	24 (12.83)	24.69 (9.21)	0.491	0.68
DT-stride-time-AI (%)	22.79 (22.91)	21.89 (17.46)	0.337	0.618
DT-stance-phase-CV (%)	16.86 (4.82)	15.95 (2.97)	0.147	0.353
DT-stance-phase-AI (%)	11.85 (7.27)	11.33 (5.8)	0.412	0.618
DT-TO-CV (%)	21.05 (10.17)	23.6 (7.98)	0.404	0.618
DT-HS-CV (%)	28.14 (9.83)	28.04 (10.35)	0.86	0.86

Bold values highlight the significant differences; Adj.P: Benjamini-Hochberg corrected *p* value; DT: Dual-task walking test; CV: coefficient of variation; AI: asymmetry index; TO: toe-off angle; HS: heel-strike angle

### The effect of anxiety on gait performance

Table 4 and the Supplemental materials displayed the effect of anxiety on gait performance. Through multiple linear regression analysis, anxiety was an independent factor of a serials of gait parameters, especially ST-TO (B = -2.599, *p* = 0.023, 95% CI (-4.82, -0.38)), ST-HS (B = -2.532, *p* = 0.024, (-4.71, -0.35)), ST-TO-CV (B = 4.627, *p* = 0.002, (1.71, 7.64)), ST-HS-CV (B = 4.597, *p* = 0.003, (1.66, 7.53)), ST stance phase (B = 1.4, *p* = 0.021, (0.22, 2.58)),and DT stance phase (B = 1.749, *p* = 0.005, 95% CI (0.56, 2.94)).

**Table 4:** Anxiety as independent variable of gait parameters

Dependent variable	B	SE	t	*P*	Lower 95%CI	Upper 95%CI
ST-stride-length	-0.073	0.028	-2.584	**0.011**	-0.13	-0.02
ST-gait-velocity	-0.114	0.032	-3.514	**0.001**	-0.18	-0.05
ST-stride-time	0.083	0.03	2.791	**0.006**	0.02	0.14
ST-stance-time	0.077	0.025	3.088	**0.002**	0.03	0.13
ST-stance-phase	1.4	0.6	2.333	**0.021**	0.22	2.58
ST-TO	-2.599	1.132	-2.296	**0.023**	-4.82	-0.38
ST-HS	-2.532	1.112	-2.277	**0.024**	-4.71	-0.35
ST-TO-CV	4.627	1.513	3.088	**0.002**	1.71	7.64
ST-HS-CV	4.597	1.496	3.072	**0.003**	1.66	7.53
DT-stride-length	-0.079	0.034	-2.328	**0.022**	-0.15	-0.01
DT-gait-velocity	-0.091	0.034	-2.672	**0.009**	-0.16	-0.02
DT-stance-phase	1.749	0.606	2.886	**0.005**	0.56	2.94

Bold values highlight the significant differences; ST: single-task walking test; DT: Dual-task walking test; CV: coefficient of variation; AI: asymmetry index; TO: toe-off angle; HS: heel-strike angle

## Discussion

This study investigated the influence of anxiety on gait performance in patients with PD by sensor-based analysis. We found that gait performance was generally worse in patients with anxiety compared to those without anxiety in both ST and DT situations. Specifically, anxiety patients experienced a significant decrease in stride length, gait velocity, TO and HS angles, as well as a significant increase in stride time, stance time, stance phase, and variability of TO and HS angles during ST walking, with these differences diminishing during DT walking. Furthermore, using multilinear regression analysis, we demonstrated that anxiety was an independent factor of various gait parameters. Our findings imply a close relationship between anxiety and gait disturbances in PD patients, with anxiety potentially impairing gait performance and negatively affecting patients’ quality of life.

### Explanation of our findings

**Fig. 2:**
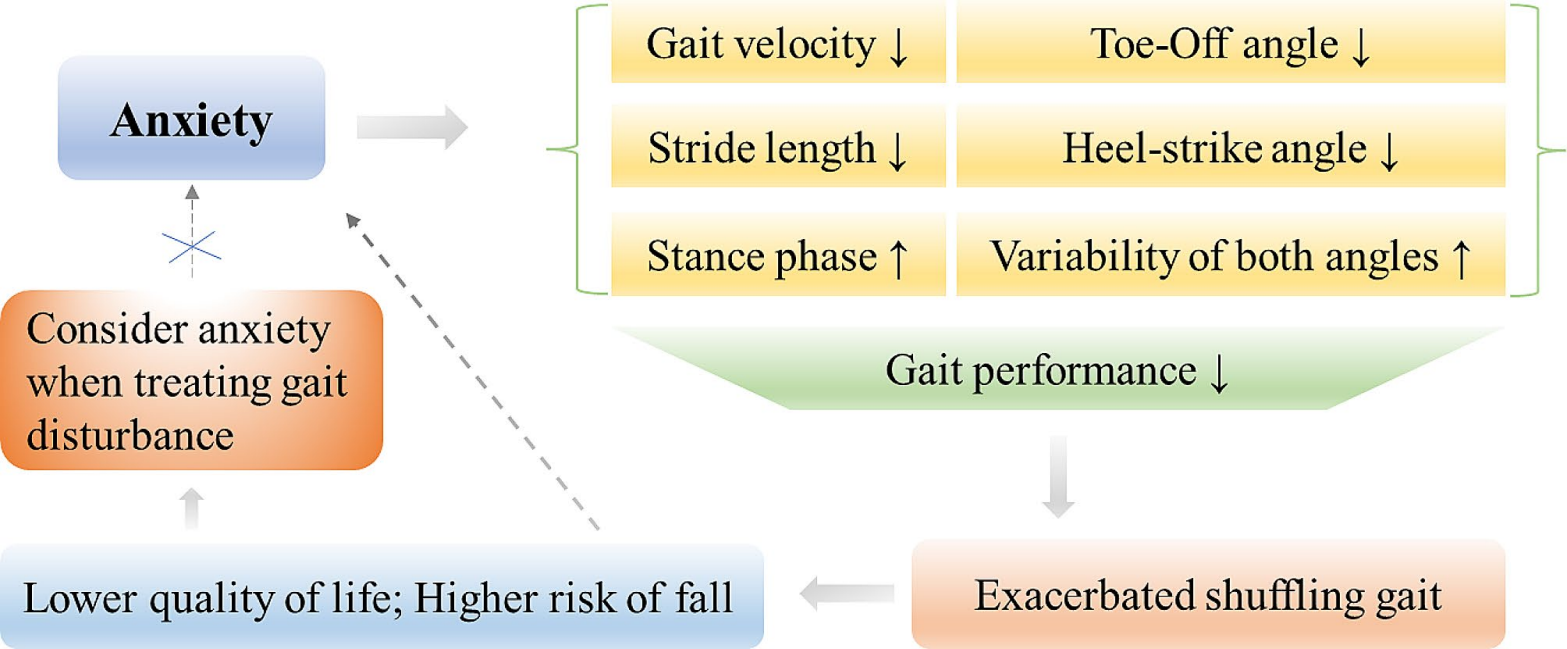
Illustration of the main finding of this study Anxiety potentially leads to impairments of gait performance, including lower gait velocity, shorter stride length, longer stance phase, smaller Toe-Off and Heel-strike angles, and larger variabilities of both angles, which exacerbates shuffling gait and results in lower quality of life and higher risk of fall, which may in turn aggravate anxiety. Our study highlights the necessity to consider anxiety as an important factor in the assessment and treatment of gait disturbance to cut off this vicious cycle

Our study adds new quantitative evidence to the existing knowledge of effect of anxiety on gait disturbances in patients with PD and extends understanding of the details and extent of this effect. According to our study, patients with anxiety displayed more pronounced bradykinesia, shuffling gait, and longer stance time than those without anxiety. This suggests that the motor abilities of PD patients with anxiety are affected during walking, which might be related to the presence of anxiety. Subsequently, the regression analysis reveals that anxiety likely leads to lower gait velocity, shorter stride length, longer stance and stride time, longer stance phase, smaller TO and HS angles, and larger variabilities of these two angles. These finding implies that anxiety potentially aggravates gait disturbances and should be considered in the process of clinical decision-making for the management of PD patients with gait disorders (Fig. 2). It is noteworthy that the regression analysis in our study reveals a considerable influence of anxiety on TO and HS angles and their variabilities, as evidenced by the absolute values of their regression coefficients being greater than 2.5. This indicates that the most prominent gait change caused by anxiety is shuffling gait and their large variations, which may lead to substantially higher risk of falls. Therefore, extra caution should be taken to prevent falls in patients who are experiencing anxiety (Fig. 2).

However, the potential mechanism of anxiety’s negative impact on gait performances is not very clear. One perspective is that anxiety may exacerbate gait disturbances by consuming resources needed to overcome or compensate for sensory-perceptual deficits in patients with PD, leading to even more severe gait impairments [18, 19]. While another perspective is that anxiety may over activate the locus coeruleus which would provoke “Supraoptimal arousal” of the network between motor and complementary sensory, limbic, and cognition regions, subsequently bring about competing inputs of these complementary networks, and finally results in aggravated gait disturbances (e.g., FOG) [20, 21]. In our study, under DT status, the difference of gait performance between PD patients with and without anxiety tends to be diminished. Accordingly, this might be due to the condition that in the non-anxious group, the complementary resources or networks are occupied by extra cognitive tasks, resulting in reduced resources available for compensating motor functions, leading to gait performance in non-anxious PD patients approaching that of the anxious group. A study comparing the ability between ST and DT conditions to identify gait markers associated with the progression of PD revealed that fewer gait parameters were with statistical differences in the DT state [22]. Although participants were instructed to focus on both tasks simultaneously, some individuals may tend to concentrate more on walking, while others may lean towards cognitive tasks, which can potentially compromise the reliability of the findings. Therefore, we acknowledge as well that when assessing the impact of anxiety on gait, the DT state, which involves cognitive task and increases the complexity of the issue, is less favorable, making the ST condition more suitable. In addition, except for ST-TO-CV and ST-HS-CV, our study did not show other increased gait variability and asymmetry in PD patients with anxiety compared with those without anxiety. This might be attributed to the fact that during gait monitoring, anxiety has a stable effect on both sides of motor and complementary networks. Another potential explanation may lie in the fact that these parameters are obtained through multiple computations rather than being raw data, resulting in less pronounced differences between the two groups.

### Filling the gap

A paucity of studies has employed sensor-based quantitative approaches to analyze multiple gait parameters in investigating the relationship between anxiety and gait in patients with PD. First, our findings are consistent with a single previous study showing that PD patients with high level of anxiety had reduced gait velocity and step length compared with both the healthy control and low-level-anxiety groups during both ST and DT conditions [23]. However, we observed an additional reduction in TO and HS angles that was not examined in this prior study and in a much larger cohort of PD patients. Our study did not involve healthy individuals as a control group, as multiple studies have already established that patients with PD generally had poorer gait performance than healthy individuals [15, 24]. Second, a few previous studies have found that anxiety was a potential contributor of FOG [25]. Compared with these studies, our study assessed dozens of other gait parameters affected by anxiety, thereby providing significant supplementary insights into the impact of anxiety on gait disturbance. Third, a single study evaluated the difference of gait performance between PD patients with and without non-motor symptoms [26]. In contrast, our study mainly focused on anxiety, as we observed an apparent decline of gait impairments in PD patients clinically when they experienced acute or chronic anxiety.

### Application

Our findings reveal that anxiety impacts gait performance in patients with PD, highlighting the need to consider this factor in the assessment and treatment of gait disturbance. Healthcare providers should consider anxiety as an important factor when treating gait disorders. This may involve assessing a patient’s anxiety symptoms and determining whether they need to be addressed as part of the treatment plan. Treatment options for anxiety symptoms may include medication [27], psychotherapy [28], exercise [29], and relaxation techniques, such as music [30], meditation [31], and yoga [32]. Addressing anxiety symptoms may not only help to improve a person’s overall mental health but also lead to better outcomes. By reducing anxiety, patients may be better able to focus on their physical therapy and rehabilitation and be more motivated to engage in activities that can improve their gait and overall mobility.

### Strengths and limitations

The main strengths of this study are: (1) By utilizing wearable sensors, we conducted a thorough investigation of gait impairments in PD patients with and without anxiety during ST and DT walking assessments. (2) Our study expanded upon prior research by analyzing alterations in additional spatiotemporal gait parameters both under ST and DT conditions. (3) By concentrating on the influence of anxiety rather than all non-motor symptoms on gait disorders, our study is better positioned to facilitate personalized treatment approaches for PD patients accompanying with anxiety.

This study has some limitations. First, given that dopaminergic therapy can impact gait performance [12], which would conceal the impact of anxiety on gait, we conducted our study in the OFF state to diminish the influence of dopaminergic treatment and uncovered the true extent of anxiety’s effect on gait. However, this approach does have limitations since PD patients are generally in the ON state during clinical treatment, and our study cannot reflect this practical scenario. Future research could explore the effect of anxiety on gait at the ON state. Second, the recruitment of participants from a single center may result in selection biases. To address this issue, future studies could pursue multi-center cooperation and enlarge the sample size. Third, the study utilizes a cross-sectional design, which limits the power to establish causation relationship between anxiety and gait impairments. Thus, future research could adopt a longitudinal design. Fourth, the current study did not include a comparison of neuroimaging data between PD patients with and without anxiety. Future research could apply such a comparison to further explore the mechanism underlying anxiety’s impact on gait. Fifth, gait speed was not utilized as a factor that could potentially confound the results.

## Conclusion

In conclusion, we showed that gait impairments are more prominent in PD patients with anxiety, particularly manifested in slower gait velocity, shorter stride length, and increased shuffling and stance phase. Regression analysis further identified anxiety as an independent influencing factor of these gait disturbances. This highlights the importance of considering anxiety in PD precision therapy and prioritizing its treatment to achieve better outcomes. Future RCTs could investigate whether treating anxiety in PD patients can significantly improve gait disturbances.

### Electronic supplementary material

Below is the link to the electronic supplementary material.

Supplementary Material 1

## Data Availability

The datasets used and/or analyzed during the current study are available from the corresponding author on reasonable request.
